# Exploring the Relationship between Surface Acting, Job Stress, and Emotional Exhaustion in Health Professionals: The Moderating Role of LMX

**DOI:** 10.3390/bs14080637

**Published:** 2024-07-25

**Authors:** Ibrahim Yikilmaz, Lutfi Surucu, Ahmet Maslakci, Alper Bahadir Dalmis, Emete Toros

**Affiliations:** 1 Department of Management and Organization, Faculty of Business Administration, Kocaeli University, Kocaeli 41380, Turkey; 2 Department of Business Administration, Faculty of Economics, Administrative, and Social Sciences, Bahçeşehir Cyprus University, Mersin 10, Nicosia 99010, Turkey; lutfi.surucu@baucyprus.edu.tr (L.S.); ahmet.maslakci@baucyprus.edu.tr (A.M.); 3 Department of Management and Organization, Aeronautical Vocational School of Higher Education, University of Turkish Aeronautical Association, Ankara 06790, Turkey; abdalmis@thk.edu.tr; 4 Faculty of Business Administration and Social Sciences, University of Kyrenia, Mersin 10, Kyrenia 99320, Turkey; emete.toros@kyrenia.edu.tr

**Keywords:** surface acting, job stress, emotional exhaustion, LMX, health professionals

## Abstract

Rapid organizational changes due to technological advancements, high-efficiency expectations, and uncertainties, particularly in healthcare, have led to a global stress epidemic among em-ployees. This has been exacerbated by the COVID-19 pandemic and evolving workplace practices. Surface acting, or the suppression and faking of emotions, significantly contributes to this stress and burnout, impacting not only individual healthcare professionals but also healthcare systems’ overall effectiveness and sustainability. Providing adequate resources in high-demand work environments is, thus, essential to mitigate these negative experiences. Leader–member exchange (LMX) can play a pivotal role in understanding and addressing the needs and expectations of healthcare professionals. Drawing on Conservation of Resources (COR), Job Demands-Resources (JD-R), Social Exchange theories, and Grandey’s Emotional Regulation Model, this study analyzed data from a convenience sample of 350 healthcare professionals. The results reveal that surface acting intensifies healthcare professionals’ experiences of job stress and emotional exhaustion. Notably, the study empirically demonstrated that high levels of LMX in healthcare professionals’ relationships with their leaders can mitigate the impact of surface acting on job stress and emotional exhaustion. These findings offer valuable insights for managers and policymakers, highlighting the importance of LMX in maintaining sustainable management practices in complex and stressful healthcare organizations.

## 1. Introduction

In 1966, “Protecting the Health of Eighty Million Workers—A National Goal for Occupational Health” by the National Advisory Environmental Health Committee to the Surgeon General of the United States (US Department of Health and Human Services) reported that employees’ stress experiences began to increase [1]. It emphasized that this threatens the mental and physical health of employees, and that technological advances and psychological demands in the workplace are very significant factors [1]. The situation has worsened since then. In today’s fast-paced world, workplaces are often stressful due to intense uncertainty and competition dynamics, too. In 2016, the World Health Organization declared work-related stress a global epidemic, and the impact of the COVID-19 pandemic is predicted to elevate these stress levels even further in 2022 [2].

Many theories about stress, such as Transactional theories of work-related stress, Cooper and Palmer’s model of work stress, Cooper and Marshall’s model of work-related stress, etc., define job stress to a certain extent [3,4,5]. Compared to the initial period, the definition of stress is not only seen as a factor that threatens the individual, such as “the general adaptation syndrome (GAS)”, but also as a tool for predicting future difficulties and environmental factors. It is discussed positively, such as adaptation to change, expectations, and eustress [6]. The evaluation of stress as a positive factor in the workplace is based on elevated arousal, which can occasionally boost performance and concentration in the short term. This concept is known as the “Yerkes–Dodson Law” [7]. However, there must be a limit. Exceeding the optimal level and accepting high stress as the new normal and performance criteria will negatively affect an individual’s health and performance in the medium and long term [8,9,10] and cause the work-related stress epidemic mentioned in the various statistics previously shared above. At this point, the National Institute for Occupational Safety and Health (NIOSH) model [11] suggests that job stress and health problems arise when job demands do not align with workers’ needs, expectations, or abilities. Work-related psychosocial factors (limited control over work pace, inadequate support from colleagues or management, and insufficient resources to complete tasks effectively) lead to various reactions affecting a person’s overall personality and health. At this point, the healthcare sector stands out among other industries due to its high workload and job demands. Stress is magnified as performance directly impacts human life. Factors such as irregular and night shifts, a higher patient volume, role conflict, and limited autonomy add to this stress. The work environment for healthcare professionals, already burdened with high workloads and job demands before the COVID-19 pandemic, has reached critical levels following the pandemic [12,13]. The demanding nature of this environment can cause an imbalance between job demands and resources among health professionals. According to the Job Demands-Resources and Conservation of Resources theories, this imbalance can lead to high stress levels and burnout [14,15]. Indeed, it was recently revealed that workplace-related stress and burnout levels among healthcare professionals reach up to 78% in developed countries, while in developing countries, they range between 40–60% [16,17]. High levels of stress and burnout experienced by healthcare professionals can have many adverse effects on public health. These effects impact not only the individuals and their organizations but also patient care. Long-term stress and burnout can result in decreased job satisfaction among health professionals, lower levels of physical and mental health, depression, and even job resignation [18,19,20,21,22,23,24,25]. Once more, adverse effects beyond the individual can lead to decreased performance in healthcare organizations, increased absenteeism, service disruptions, and, ultimately, harm to patients and the entire healthcare system [14,26,27,28,29]. Indeed, healthcare professionals’ well-being directly impacts the healthcare system and patient welfare [30]. Addressing stress and burnout among healthcare professionals is essential, as they can increase the error rate in medical procedures, cause safety issues, and potentially elevate patient mortality rates [26,31]. Moreover, when healthcare professionals’ decisions to quit their jobs due to exhaustion and frequent disruptions are factored in, this situation alone reportedly costs the US healthcare system more than USD 4 billion [26,32]. In another study conducted in Canada, it was reported that the trend of healthcare professionals retiring early, coupled with a shortage of physicians in certain specialties, could lead to patient service losses of up to USD 160 million [33]. This negative situation, characterized by a heavy workload and stressful working environment, will have more devastating effects, especially in Turkey. The latest OECD 2023 report ranks Turkey among the bottom five countries, with only 2.2 doctors and 2.8 nurses per 1000 population. These numbers are significantly below the OECD average of 3.7 doctors and 9.2 nurses [34]. Considering all these adverse conditions collectively, the stress and burnout experienced by healthcare professionals is a significant issue that needs highlighting. Identifying the contributing factors and developing solutions to prevent such problems to sustain public health and the system effectively is essential.

Emotional labor is a crucial factor contributing to high levels of stress and burnout in the workplace. For health professionals, this requires greater emotional involvement compared to other professions. Indeed, health professionals do not just perform their duties using technical knowledge related to their expertise, but they also utilize their communication and relationship skills. These skills are equally important and influential as their technical knowledge [35,36]. Certain characteristics of the work environment, such as high job demands, significantly influence the emotional labor experience of healthcare professionals. They often have to manage their emotions while communicating and building relationships, all within the scope of their work requirements [37,38]. Emotional labor refers to regulating and managing an employee’s emotions in line with job demands [37]. Essentially, two emotional labor strategies are demonstrated: surface acting, where emotions not actually felt are displayed in line with job requirements and social interaction, and deep acting, where the displayed emotion is internalized as a genuine feeling. Particularly in the context of an emotional-regulation strategy, an employee may exhibit an emotion they do not truly feel (surface acting), thus creating emotional cognitive dissonance. The ongoing burden of such insincere emotion (surface acting) can cause stress and burnout, triggering various other negative workplace experiences [37,39,40,41,42,43]. In the complex, multi-actor communication that healthcare professionals engage in with patients and their relatives, they are obligated to provide care, treatment, psychological support, and a hopeful approach. This often leads them to arrange their genuine responses within the confines of their professional duties, thus exhibiting surface acting [44,45,46,47]. This situation leads to healthcare professionals exhausting their resources and experiencing high levels of stress and burnout, in accordance with JD-R and COR theories [36,48,49,50]. This triggers a mechanism that jeopardizes healthcare professionals and the entire healthcare system. Consequently, finding solutions to mitigate the effects of the mechanism linking emotional labor, stress, and burnout becomes a pressing issue. One contemporary approach that could potentially offer a solution to this problem is the concept of Leader-Member Exchange (LMX). The LMX theory emphasizes the importance of the relationships between leaders and their team members. By improving these relationships, leaders can provide a supportive framework that can help their team members manage the emotional job demands of their jobs more effectively. According to J D-R and COR theories, leaders’ high-quality relationships are valuable resources, contributing to the gain of information and tangible assets, and the horizontal communication established by Leader-Member Exchange (LMX) could be vital in mitigating the adverse effects on employees due to emotional labor, stress, and burnout [51,52,53].

Numerous studies cite stress and burnout as primary contributors to negative workplace experiences. While much research has been conducted on their antecedents, there is insufficient emphasis on the role of the leadership process [54,55]. When examining the relationship between emotional labor, stress, and burnout, it is important to note that the variables considered are often limited to the work environment (e.g., compensation) and certain psychosocial factors (e.g., psychological capital, emotional intelligence) [56,57,58]. At this point, examining the moderation of LMX, especially on the interaction mechanism between emotional labor, stress, and burnout, based on the combination of JD-R, COR, and Social Exchange Theories, may offer a solution regarding the intrinsic motivation, resources, and support healthcare professionals need. Moreover, the existing literature suggests that job demands and resources may vary based on job characteristics and cultural tendencies toward emotional management. It also indicates that the relationship between emotional labor stress and burnout can be broader, affecting all healthcare professionals. This includes not just nurses but also doctors, patient relations staff, secretaries, and others. It may also be influenced by the characteristics of a collectivist society. There are calls to examine these aspects using a sample encompassing all these issues [48,56,57,58,59]. This study aimed to address the existing gaps in the literature by examining the moderating effect of Leader–Member Exchange (LMX) on the impact of healthcare professionals’ surface-acting emotional-labor strategies on job stress and emotional exhaustion. The study’s design first details the relationship between the existing literature on surface-acting, job stress, emotional exhaustion, and the potential role of LMX. Subsequently, the research model and methodology are based on existing literature. Finally, the study’s findings and their theoretical and practical implications are discussed. The study’s results highlight the adverse interaction between surface acting, job stress, and emotional exhaustion. By considering Leader-Member Exchange (LMX) as a necessary resource and support for healthcare professionals, the study contributes significantly to the literature in the context of Conservation of Resources (COR), Social Exchange, and Job Demands-Resources (J-D-R) theories. Furthermore, examining all healthcare professionals comprehensively broadens the literature. It proposes a collective, culturally-oriented emotional management strategy in response to calls within the literature and offers valuable recommendations for sustainable public health management to policymakers and senior managers.

## 2. Literature Review

### 2.1. Surface Acting, Job Stress and Emotional Exhaustion

The workplace environment is not just a significant social area where employees spend much of their time but also serves as a stage for their behavior. Here, employees continually strive to improve themselves, influence customers, and interact with colleagues. The balance between job demands and an employee’s resources determines job performance and satisfaction. However, an imbalance can have detrimental effects on both the service recipient and the employee. This predicament can be explained by the Job Demands-Resources (JD-R) theory and the Conservation of Resources (COR) theory. J D-R theory suggests high job demands and low resources lead to a highly stressful work environment. On the other hand, COR theory states that an individual is continually trying to protect and balance their resources. When these resources are depleted in the workplace, and the individual cannot replace them, it leads to negative experiences. Both theories emphasize that when a work environment fails to replenish the resources spent due to high job demands, it results in resource deprivation and negative work experiences. Therefore, it’s crucial to monitor and understand an individual’s needs in the workplace accurately. Additionally, measures should be taken to optimize resources, i.e., providing each employee with the right amount of job resources to maintain an optimal resource level [60,61]. However, not all organizations can provide the perfect balance of job demands and resources to their employees, and not all employees have unlimited resources. This imbalance can lead to significant stress and burnout. Work-related stress arises from negative emotions experienced during work processes, threatening work conditions, and unmet job demands [62,63,64]. Burnout can be described as a state of being “mentally, physically, and emotionally” drained [40,65]. It is characterized by three main aspects: emotional exhaustion (feeling emotionally exhausted and depleted), depersonalization (developing a detached or cynical attitude towards others), and diminished personal accomplishment (experiencing a decline in the sense of personal achievement or effectiveness in one’s profession). Stress and burnout are significant issues faced by healthcare professionals. They are especially prevalent in hospital settings due to high job demands, leading to numerous negative situations [66,67,68]. Healthcare professionals often find themselves in high-stress situations due to the demanding nature of their hospital jobs. The workload is typically heavy and includes irregular and night shifts, a large patient volume, role ambiguity, and significant responsibility for patient outcomes. Additionally, a lack of autonomy and the emotionally taxing nature of dealing with suffering and death contribute to the stress [69,70]. Long-term stress and burnout can adversely affect the quality of care provided and also negatively impact the professionals’ health and satisfaction, leading to physical and mental health issues, increased turnover, and absenteeism [17,68,71,72]. Stress and burnout not only jeopardize the physical and mental health of employees, but they also negatively impact patients and the efficiency of the healthcare system [26,31]. These adverse effects can lead to a decline in patient contentment, a rise in medical inaccuracies and safety incidents, and an increase in patient death rates. It is believed that burnout drains more than USD 4 billion annually from the U.S. healthcare system. This is due to substandard medical treatment and the fallout from medical staff leaving their jobs or being absent [26,32]. At this point, it is emphasized that understanding the causes of stress and burnout, as well as proposing strategies to minimize their negative effects, is an important matter [26,32].

When it comes to burnout and stress, emotional labor comes to the fore as another negative workplace experience that significantly strains healthcare professionals’ resources. Emotional labor was initially defined as a strategy for managing an individual’s emotions for societal interaction through the use of facial and physical expressions [73]. In later studies, emotional labor was viewed as a task that employees are expected to perform in the workplace. It is considered a performance criterion, even if employees do not genuinely experience certain emotions in their work environment [37,39]. The emotions an employee is expected to display during their work can vary. They may need to appear sincere, excessively serious and professional, or cheerful, even when experiencing complex and negative emotions. Since the relationship between an organization, its employees, and its customers is a social interaction, the behaviors expected from an employee during service delivery are defined as emotional labor [74]. This refers to a process of commodification that minimizes negative impacts, emphasizes positive ones, and manages emotions to meet social and professional obligations [75,76]. Emotion regulation theory, addressed in this process, suggests that individuals can control when they experience emotions, which emotions they experience, and how they express them. This theory also applies to employees’ emotional control [77]. This process takes place in two approaches: antecedent-focused emotion regulation and response-focused emotion regulation [77,78]. Antecedent-focused emotion regulation refers to a mental tactic applied at the initial stage of an emotional response. This approach entails altering the circumstance or the individual’s understanding of the circumstance to handle better and regulate their emotions. Response-focused emotion regulation involves an individual modulating the expression of their emotional response, thereby influencing their physiological, experiential, or behavioral reactions. Due to the current situation and expectations, the individual strikes a balance between their internal emotions and the emotions they express. This balance results in anticipated behavior. In the existing literature, these emotional labor strategies are generally classified as surface-acting and deep-acting [37]. Surface acting is defined as an employee adopting an emotional-regulation strategy, exhibiting expected behavior for their role even when they do not genuinely feel it. On the other hand, deep acting is when an employee adopts the displayed emotion as a natural emotion. A key issue arises when employees display emotions they do not truly feel, leading to the depletion of their physical and psychological resources. When examining the existing literature, it is apparent that surface acting, as an emotion-regulation strategy, can result in many negative experiences for both the employee and the organization. Long-term surface acting can cause employees to experience burnout, stress, job tension, insomnia, and increased absenteeism [79,80,81]. It is also emphasized that human resources, an essential and strategic resource for businesses, can cause them to leave their jobs [82,83] and prevent them from working effectively and efficiently in the long term [42].

Surface-acting emotional labor strategy has a more negative impact on individuals and organizations than deep acting, and in many studies, its effect on burnout and job stress stands out as the primary source of other negative experiences [39,40,41,42]. This situation is particularly evident when the employees are healthcare professionals. Due to the multidimensional nature of their work, healthcare professionals play a crucial role. They not only provide direct care to patients but also coordinate and communicate operational processes. Additionally, they establish complex emotional connections with patients, their relatives, and various segments of society. Despite navigating these complex emotional interactions and experiencing numerous negative emotions, they often uphold their professional duties due to their organizational commitment and strong sense of duty [44,47]. The emotional labor pressure on healthcare professionals increases when factors like rising competition, operational effectiveness, efficiency concerns, high patient satisfaction, and volume are added to this emotional burden [45,46]. At this point, the Job Demands-Resources (JD-R) and Conservation of Resources (COR) theories suggest that employees need to maintain a balance of resources to prevent job demands from causing resource loss and deprivation. Healthcare professionals may experience high levels of stress and burnout if they continually suppress their personal experiences and emotions. Trying to maintain an always hopeful, motivated, energetic, supportive, objective, and self-sacrificing demeanor with their patients can lead to resource depletion. The existing literature indicates that healthcare professionals’ exposure to surface acting as an emotional regulation strategy increases job stress [48,49,50,84,85] and burnout [36,50,86]. Burnout is typically discussed in three stages. The first stage, emotional exhaustion, is often the most noticeable and commonly experienced aspect of burnout [87]. Significantly reducing emotional exhaustion, the initial burnout stage, can prevent its adverse effects [87,88]. Emotional exhaustion depletes an individual’s physical and psychological resources, often leading to feelings of being overwhelmed. In the context of cognitive dissonance theory, when an employee displays an emotion that does not align with their true feelings (surface acting), it results in emotional and cognitive dissonance [37,43,73]. The stress and pressure from this situation can lead to emotional exhaustion [89,90,91].

The literature indicates that the link between emotional labor and burnout is typically addressed within the retail sector. For more comprehensive results, it is crucial to examine this relationship in other professions with high job demands [92,93]. Due to the high demands and caregiving nature of their profession, many healthcare professionals may not realize they are experiencing emotional labor. This is often treated as a job requirement in healthcare institutions, and despite its negative consequences, the issue is not adequately addressed [94]. Although interest in the emotional, cognitive, and physical load on healthcare professionals has grown in recent years due to heightened workplace stress, comprehensive research is still needed [46]. Moreover, regarding healthcare professionals, studies generally examine the relationship between emotional labor, stress, and burnout in nurses. There is limited research on other healthcare professionals [48,56,57,58,59]. It is also important to note that emotion and cognition can vary across cultures. A study reveals a variance in the interaction between stress and emotional labor among nurses, depending on whether they come from individualistic or collectivistic cultures [95]. Owing to these cultural differences, there is a demand for more research, particularly in collectivist cultures. This would help yield accurate and comprehensive results on the mechanism of emotional labor, leading to job stress and emotional exhaustion [59,96]. Based on the theories of Emotion Regulation, Job Demands-Resources (JD-R), and the Conservation of Resources (COR), the following hypotheses were formulated to determine how surface acting, job stress, and emotional exhaustion that health professionals experience in the work environment due to high job demands affect employees’ resources and the results of their efforts to regulate emotions. An inclusive sample consisting of various healthcare professionals, such as doctors, nurses, and secretaries, within a collectivist culture was targeted.

**H1.**  *The surface-acting strategy increases job stress levels experienced by healthcare professionals.*

**H2.**  *The surface-acting strategy increases emotional exhaustion levels experienced by healthcare professionals.*

### 2.2. LMX As a Moderator

Work relationships involve continuous mutual interactions [97]. In social exchange relationships, often referred to as “reciprocal interdependence” [98], there is a mutual dependency. While it is understood that leaders significantly influence the quality of Leader-Member Exchange (LMX) relationships, followers also play a vital role in shaping these relationships [99,100]. This perspective differs from traditional leadership models, which predominantly focus on the direct influence of leader traits and actions on follower attitudes and behaviors.

The Leader-Member Exchange (LMX) theory initially focused on role theory but has adopted more of the social exchange theory over time [101,102,103]. Low-LMX relationships are primarily economic, where the exchange of tangible benefits like performance-based rewards is immediate and balanced. However, high-LMX relationships are more socially oriented, characterized by mutual responsibility and reciprocity [104,105,106]. The shift from economic to social exchange in these relationships enhances elements of loyalty, commitment, support, and trust [98,107]. This mutual reciprocation in high-LMX relationships cultivates an increased emotional bond between leaders and followers [97,107,108].

The intense emotional bond, trust, respect, influence, and adequate and correct amount of information that employees develop with their leaders in a work environment, as posited by social exchange theory, lays the groundwork for displaying more positive role behaviors related to their jobs [105,109,110]. It was emphasized that the horizontal communication and interaction established by LMX play an essential role in reducing the adverse effects experienced by the employee due to the relationship between emotional labor, stress, and burnout [52,53].

The Leader-Member Exchange (LMX) consists of three stages: role-taking, role-making, and routinization [111]. These stages provide a critical understanding of how to mitigate the impact of surface acting on job stress and burnout. In the role-taking phase, a supportive leader can reduce emotional stress from surface acting, thus avoiding early tendencies towards burnout and stress. As team members move to the role-making stage and rely more on their leader, compassionate leaders can identify and relieve the emotional burden, offsetting potential burnout and job stress. When routinization comes into play, the strong relationship between leaders and team members is a constant safeguard against the negative aspects of emotional labor, ensuring that neither form results in extreme burnout or job stress. If the LMX process is properly implemented, it fosters supportive in-groups among employees and superiors, leading to more effective stress management and reduced emotional labor. According to the Job Demands-Resources (JD-R) theory, leaders can be seen as valuable resources when their high-quality relationships contribute to the gain of information and tangible assets [51,102].

The concept of the Leader-Member Exchange (LMX) is rooted in role theory, which forms the bedrock of the role negotiation process inherent in the LMX relationship and is instrumental in reported role stress [112]. Supervisors can help subordinates navigate their roles effectively by clearly defining roles and expectations. Supportive and communicative high-LMX relationships may help reduce uncertainties and ambiguities [113]. As such, high-LMX employees often have positive role perceptions that closely align with their supervisors’ expectations [112]. Conversely, low-LMX employees may experience more stress due to a lack of information or limited support. Existing studies show that individuals assess their standing by comparing themselves with peers and observing the LMX distribution in their workgroup [102]. Those in low-LMX relationships may see it as an added role stressor, treating it as a demand rather than a resource. Thus, it is suggested that there is an inverse correlation between LMX quality with job stress and burnout.

**Figure 1:**
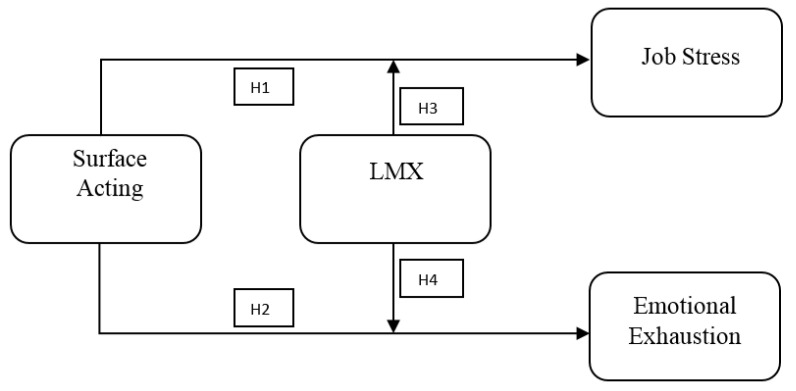
Research model.

Although stress and burnout in the workplace are considered precursors to negative experiences in many workplaces, it is emphasized that the leadership process is not sufficiently focused during the solution phase [54,55]. Additionally, while job demands and resources play an essential role within the organization, they may vary based on specific job characteristics. When the extant literature is examined, burnout and job stress research has mainly focused on determining the prevalence of burnout and stress and understanding the impact of demographic and organizational characteristics on these variables. However, there is a research gap in terms of addressing burnout and job-stress management from an organizational perspective, especially in terms of investigating the impact of emotional labor on burnout and stress in leader–member relations among healthcare professionals (not only nurses, but also doctors, secretaries, etc.) in a comprehensive manner. Certain work environment characteristics (e.g., compensation or role) and certain psychosocial variables (e.g., psychological capital, core self-evaluations, social and emotional intelligence) are generally considered moderator variables [56,57,114]. The sole study available during the literature review determined that LMX moderated the impact of emotional labor on burnout, thereby reducing nurses’ negative experiences [115]. However, it is acknowledged that the study has limitations in understanding the relationship between emotional labor, burnout, and Leader-Member Exchange (LMX) due to its small sample size of 165 nurses and focus solely on nurses. More research on this subject is needed. In this context, the aim is to investigate the moderator role of LMX in mitigating the impact of surface acting, which results from high job demands experienced by healthcare professionals on job stress and emotional exhaustion. This is intended to contribute to developing sustainable public health management practices. Based on the existing literature and theories of Emotion Regulation, Job Demands-Resources (JD-R), and the Conservation of Resources (COR), the following hypotheses were formulated to examine the effect of LMX as an essential organizational resource in reducing negative workplace experiences, such as surface acting, job stress, and emotional exhaustion, which deplete the resources of healthcare professionals in a high-demand environment. The research model is depicted in Figure 1.

**H3.**  *LMX plays a moderating role in the effect of surface acting on job stress.*

**H4.**  *LMX plays a moderating role in the effect of surface acting on emotional exhaustion.*

## 3. Materials and Methods

### 3.1. Research Purpose and Sample

The study’s goal was to examine the impact of surface acting emotional labor strategy on work stress and emotional exhaustion and how leader–member exchange moderates this relationship. Data were gathered through surveys for this purpose. Upon deciding to conduct the study, ethical permissions were granted from the Cyprus World Peace University Ethics Committee (WPU-ETK-2023-10). The study’s participants were hospital employees. The chief physicians at Ankara hospitals were approached for permission to conduct the study. Two hospitals granted permission. Trained interviewers were assigned to these hospitals, and surveys were administered on-site to participants chosen through convenience sampling (n = 350).

The survey was divided into four sections. The first included a demographic information form, the second featured a surface acting emotional labor scale, the third contained a job-stress scale, the fourth had an emotional-exhaustion scale, and the final section included a leader–member exchange scale. The survey consisted of 39 total statements.

### 3.2. Measurement Tools

The scales used in this research have been previously tested for validity and reliability. They are also commonly used in past health sector research [116,117]. Detailed information about these scales is provided below.

Surface Acting: The surface-acting sub-dimension of the emotional labor scale developed by Diefendorff, Croyle, and Gosserand (2005) is used to measure the participants’ emotional labor levels [118]. This scale was based on the emotional labor scales of Grandey (2003) and Kruml and Geddes (2000) [119,120]. The Turkish validity and reliability study for this scale was conducted by Basım and Beğenirbaş (2012) [121]. The scale contains six statements, and participants rated these statements on a scale from 1 (Never) to 5 (Always). Sample items on the scale include “I play a role in taking proper care of my patients” and “I wear a mask to display the emotions required by my profession”.

Job stress: The job-stress scale, developed by House and Rizzo (1972), was utilized to gauge participants’ perceptions of job stress [122]. The scale’s validity and reliability in Turkish were verified by Efeoğlu (2006) [123]. The scale consists of seven statements, with participants rating each item on a scale of 1 (Strongly Disagree) to 5 (Strongly Agree). Examples of statements include “Problems related to my job cause me to have sleep problems” and “I work under a fair amount of tension”.

Emotional Exhaustion: The emotional exhaustion dimension of the Maslach Burnout Inventory, developed by Maslach and Jackson (1981), was used to assess the participants’ levels of emotional exhaustion [124]. The validity and reliability of this scale in Turkish were confirmed by Ergin (1992) [125]. This scale consists of nine statements. Participants responded to these items on a scale ranging from 1 (Does Not Describe at All) to 5 (Describes Completely). Sample statements from the scale include: “I feel frustrated with the work I do” and “I feel emotionally drained from my job”.

Leader–Member Exchange: The Leader–Member Exchange (LMX-MDX-12) scale, developed by Liden and Maslyn (1998), was utilized to assess the participants’ interaction level with their leaders [126]. The Turkish validity and reliability study of this scale was conducted by Baş, Keskin, and Mert in 2010 [127]. The scale consists of 12 statements. Participants responded to these items on a scale from 1 (Completely Disagree) to 5 (Completely Agree). Examples of the scale’s items include: “My superior likes me as a person” and “My superior respects my job knowledge and abilities”.

Control Variables: Existing empirical literature highlights that certain demographic variables, like gender and marital status, influence emotional labor. Hence, they should be controlled in analyses [128,129]. In light of this literature, gender and marital status were identified as control variables for the analyses.

### 3.3. Analyzes

The analyses were conducted using AMOS 22 and SPSS 27 programs.

#### 3.3.1. Descriptive Analyzes

Frequency analysis was performed to determine the demographic structure of the participants.

#### 3.3.2. Primary Analysis

Reliability analyses were conducted to determine the reliability of the scales used in the research. Cronbach’s alpha coefficient and composite reliability values were considered in the analyses. Correlation analysis was performed to determine the relationship between variables.

#### 3.3.3. Hypothesis Testing

Bootstrap-based regression analysis (Process Macro-Model 1) was used to test the research hypotheses. This method provides stronger statistical results than other methods [130]. Therefore, it is frequently used in recent studies [131]. To obtain more reliable results, bootstrap-based regression analysis (Process Macro-Model 1) was used in this study.

## 4. Results

Table 1 displays the demographic characteristics of the 350 study participants.

Upon examining the demographic characteristics of the participants, it was found that 209 were women and 141 were men. Most of these participants were married (64.3%) and held a bachelor’s degree (44.9%). Regarding age, 55 participants were 25 years old or under, 122 were between 26 and 35 years old, 115 were between 36 and 45 years old, and 58 were over 45 years old. Professional roles among participants included 50 physicians, 77 nurses or midwives, and 36 technicians.

### 4.1. Reliability and Validity of Scales

Before the correlation and regression analyses, the validity and reliability of the study’s scales were checked. The Cronbach Alpha coefficient and composite reliability values were considered in the scales’ reliability tests, and discriminant and convergent validities were considered in the validity control. The results are presented in Table 2.

The analysis conducted to determine the reliability of the scales (Table 2) revealed that the Cronbach Alpha value ranged from 0.806 to 0.931, and the Composite Reliability (CR) value ranged from 0.814 to 0.946. Literature suggests that the scales are reliable if Cronbach Alpha and CR values exceed 0.7 [131,132,133]. The results in Table 2 confirm the reliability of the scales. This is further supported by the Average Variance Explained (AVE) values exceeding 0.5 [134].

The final decision on construct validity was determined through discriminant and convergent validity tests. Discriminant validity was evaluated using the Fornell–Larcker criterion, with values of 0.729, 0.761, 0.740, and 0.773 (as shown in parentheses in Table 3). According to this criterion, these values should be higher than the correlation between the variables [134]. Another indicator of discriminant validity is correlations between factors being below 0.85 [135]. Both conditions are satisfied as seen in Table 3. Convergent validity requires the AVE value to exceed 0.50, and the CR value to be greater than the AVE value (CR > AVE; AVE > 0.5) [132]. Table 2 shows that these conditions are met. Lastly, the fit indices of the research model were examined. The model was found to have good fit indices (CMIN/df = 2.889, GFI = 0.954, NFI = 0.964, CFI = 0.974, RMSEA = 0.051).

### 4.2. Hypothesis Testing

The Process Macro, developed by Hayes (2017), was utilized to examine if leader–member exchange moderates the impact of surface acting on job stress and emotional exhaustion [130]. This method is often preferred in recent studies as it yields more accurate results compared to the traditional Sobel test [131]. Therefore, the Process Macro was chosen for hypothesis testing (Model 1). In the analyses conducted within the 95% confidence interval, the bootstrap value was set to 5000.

Tabachnick and Fidell (2007) recommend checking for multicollinearity before applying this analysis [136]. Following their recommendation, Variance Inflation Factor (VIF) and tolerance values were examined to determine if multicollinearity existed. The fact that the VIF values for all research variables are less than 5 (min = 1.120; max = 1.886) and the Tolerance values are greater than 0.10 indicates that there is no multicollinearity in this study [137]. The results of the analyses conducted for the hypothesis tests are presented in Table 4.

As seen in Table 4, surface acting has a positive effect on job stress (β = 0.425, 95% CI = [0.138, 0.437], t = 1.589, *p* < 0.05). Additionally, it was determined that the effect of surface acting on emotional exhaustion was positive (β = 0.296, 95% CI = [0.217, 0.244], t = 1.181, *p* < 0.05). In light of these findings, H1 and H2 were supported.

The study examined the moderating role of leader–member exchange in the effect of surface acting on job stress and emotional exhaustion by checking the significance of interactional terms. The results revealed that leader–member exchange does moderate the effect of surface acting on job stress (β = −0.271, 95% CI = [−0.191, −0.078], t = −3.581, *p* < 0.05) and emotional exhaustion (β = −0.171, 95% CI = [−0.222, −0.157], t = −3.248, *p* < 0.05). This conclusion is based on the fact that the effect’s lower and upper confidence intervals do not include zero [130]. To further understand the moderating effect of leader–member exchange on the relationship between surface acting, job stress, and emotional exhaustion at low and high levels (−1/+1 SD), a simple slope regression plot was constructed following the method suggested by Aiken, West, and Reno (1991) [138].

**Figure 2:**
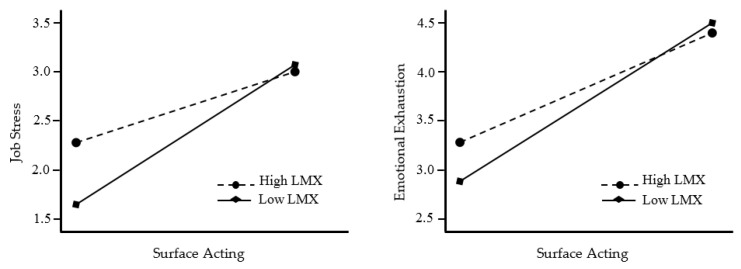
The moderating role of leader–member exchange.

According to the analysis results, Figure 2 allows the moderator role of leader–member exchange to be seen visually. Accordingly, surface acting affects job stress and emotional exhaustion more in employees with low leader–member exchange than in employees with high leader–member exchange. In other words, as leader–member exchange increases, the effect of surface acting on job stress and emotional exhaustion decreases. In line with these results, Hypothesis 3 and Hypothesis 4 were supported.

## 5. Discussion

This study examined the impact of surface acting, a strategy employed in emotional labor, including on job stress and emotional exhaustion. Leader–member exchange (LMX) was evaluated as a moderator of this effect. The findings of the study are as follows:

The study empirically demonstrated the positive effect of surface acting on job stress (H1) and emotional exhaustion (H2). Numerous research has identified that factors such as emotional strain, interpersonal conflicts, and heavy workloads significantly contribute to work-related stress [14,139,140]. JD-R and COR theories suggest that individuals strive to balance their resources with the resources expended due to their expectations. An imbalance between job demands and resources can lead to negative experiences for the individual. From this perspective, the study’s results indicate that healthcare professionals, under high job demands, display emotions they do not genuinely feel due to their responsibilities. In other words, they adopt a surface-acting emotional labor strategy. This approach significantly drains their resources and increases their work-related stress and emotional exhaustion. The employee can handle job demands and maintain high performance by ensuring a balance between daily job demands and resources [141]. Furthermore, the cognitive dissonance theory highlights that surface acting, or displaying an emotion not genuinely felt by the employee, can lead to stress. This emotional regulation through gestures and facial expressions can increase individual stress and potentially lead to emotional exhaustion [37,43,73]. Additionally, concealing one’s genuine emotions, a behavior known as surface acting, can negatively impact self-esteem and self-belief [142]. Such deliberate alterations of feelings, thoughts, and actions require substantial cognitive effort [143]. This energy expenditure can heighten stress levels and potentially harm overall well-being due to resource depletion [144]. Furthermore, hiding real emotions does not alter them but leads to less social interaction, thereby reducing social resources [145]. In addition to the challenging conditions, healthcare professionals often face negative workplace dynamics, such as verbal or physical attacks. This further depletes the balance between job demands and resources. Due to limited supportive job resources, healthcare professionals often resort to surface acting when dealing with patients. Even in developed countries like the UK (with 10–16% report rate) and the USA (with up to 50% report rate), there are high instances of injuries, verbal abuse, and assault attempts against healthcare professionals. As one of the leading “caring jobs” professions, they are frequently subjected to verbal and physical attacks [146,147,148,149,150]. When focusing specifically on Turkey, it is reported that health organizations are rapidly moving away from a safe environment, and the level of threat is increasing [151]. A report published by the Health Union (2023), a detailed report on violence in health organizations, emphasizes that the incidents increased by 31% compared to last year, with 249 violent incidents [152]. The report underlines that 422 healthcare professionals were victims of violence and shares that 85% of the incidents occurred directly during hospital examination and that the victims were primarily doctors (35%), healthcare professionals (28%), and nurses (18%). The fact that the work performed by healthcare professionals directly affects human health, their decisions or contributions to the decision-making process directly affect the individual’s life, long working hours, the threat of violence and fear, and the fact that healthcare professionals continue their service delivery processes despite everything by adopting surface acting directly pave the way for them to experience high stress and burnout. Once more, the results from this study corroborate the findings of previous research conducted on a focused group of healthcare professionals. Two studies focused on nurses determined that surface acting positively influenced stress and burnout [36,115,153]. In a comprehensive study of all hospital employees in Korea, it was reported that interpersonal service workers, in particular, exhibited more surface acting than non-ISWs, leading to increased job stress [48]. Consequently, the stressful working conditions, high job demands leading to a lack of resources, and emotional dissonance caused by surface acting contribute to extreme stress and burnout among healthcare professionals. This situation is a pressing issue that requires emphasis.

The study investigated the link between surface-acting, stress and emotional exhaustion and assessed whether the leader–member exchange (LMX) could alleviate this detrimental cycle. The results indicate that LMX can indeed moderate the adverse effects of surface acting on stress and emotional exhaustion. In particular, the study found that interactions between healthcare professionals and their managers or leaders can reduce job-related stress and emotional exhaustion. The LMX theory suggests that a distinct, unique bond exists between a manager and each team member. These individual relationships can differ significantly in their nature. A high-quality LMX relationship is marked by increased mutual trust, admiration, communication, and backing [54]. Research in the healthcare sector suggests that supervisors who foster strong relationships with their employees can help reduce their emotional exhaustion thanks to the social support and reduced role stress they provide [54]. Similarly, it has been observed that employees experience less burnout when their leaders offer them a significant amount of supportive behavior and a greater level of autonomy [154]. Higher levels of support from leaders often make employees feel obligated to demonstrate positive behaviors and perform efficiently [155,156]. Within the leader–member exchange framework, the leader’s trust in subordinates, empowering them to contribute to decision-making processes and providing autonomy, can enhance the business environment. These positive steps and establishing mutual relationships provide members an advantage in increasing their resources and balancing job demands [157,158]. This was achieved by enhancing the impact of emotional regulation strategies in the profession and diminishing the influence of emotional suppression. Indeed, the current study’s findings are consistent with the existing literature. For instance, a study conducted on 2708 Brazilian doctors found that high Leader-Member Exchange (LMX) may reduce physician burnout by mitigating perceived psychosocial job demands and providing social support [159]. Another study with nurses showed that LMX moderated the relationship between surface acting and emotional exhaustion, acting as a beneficial intra-organizational resource [115]. Furthermore, a study involving healthcare professionals and other caregivers found that surface-acting heightened emotional exhaustion, which in turn increased the need for recovery [92].

## 6. Conclusions

In healthcare organizations, the fast pace, competition, and growing diversity of patient expectations and volumes increase job demands. This often depletes the organizational resources that healthcare professionals need for effective service delivery. The study found that healthcare professionals frequently employ surface-acting emotional labor strategies in high-demand, low-resource environments, leading to heightened stress and emotional exhaustion.

Healthcare professionals, already under significant mental pressure, endure additional strain from long shifts, high-stakes performances, and occasional verbal or physical attacks. Continued exposure to surface acting can have long-term, irreversible effects on the organization and the employee. However, the study discovered that the Leader-Member Exchange (LMX) can lessen this negative impact. Positive workplace resources associated with LMX, such as trust in leaders, open communication, autonomy, and a sense of social belonging, can counterbalance the effects of surface acting. These elements balance job demands and resources, alleviating stress and emotional exhaustion. Therefore, LMX is a crucial tool for reducing the stress and emotional exhaustion experienced by healthcare professionals.

Policymakers and managers need to focus on practices that enhance the positive impact of LMX. They should pay attention to the high levels of stress, emotional exhaustion, and surface acting experienced by healthcare professionals. This focus is crucial for sustainable public health and health management.

## 7. Theoretical and Practical Implications

The study provides theoretical and practical insights for researchers and professionals in health industry management. As theoretical insights, first, the study extends the emotional labor literature by investigating the effect of surface acting on employee job stress and emotional exhaustion. This research strengthens Grandey’s Emotional Labor Model by reinforcing the significant impact of Emotional Labor (EL) on stress and emotional exhaustion. In the extant literature, there are calls that the studies between emotional labor, stress, and burnout should be examined more in other professions defined as caregiving jobs instead of the retail sector [92,93]. In addition to these calls, it is seen that the emotional labor model is conducted on samples of mostly nurse participants, with a limited number of participants in the health sector, which ranks first among caregiving professions. There are calls to examine the effects and results of the emotional labor model in collectivist societies, considering individual and cultural differences in the process of managing emotions and feelings, because the model’s effects and consequences have been studied extensively, especially in individualistic Western societies [59,96]. As a matter of fact, a study in the literature emphasizes that this difference clearly occurs, and that more studies need to test the model to understand this issue [95]. Considering all these calls in the literature, the current study expanded the literature by examining the relationship between emotional labor, job stress, and emotional exhaustion in a collectivist sample of society members, including all healthcare professionals (doctors, nurses, midwives, health secretaries, etc.). Secondly, the study extends the COR and J D-R theories literature by investigating the effect of surface acting on employee job stress and emotional exhaustion. It has been empirically demonstrated on an inclusive sample in organizations requiring high job demands, such as healthcare professionals. That failure to balance job demands and resources in environments requiring high job demands triggers a state of resource deprivation where the employee may exhibit stress and burnout. Based on COR and JDR theory, it has been revealed that the effect of surface acting on job stress and emotional exhaustion can be reduced with social support from the leader. Leader–member exchanges (LMX) are significant in providing social support at work, as they offer resources that can mitigate the adverse impact of work demands like surface-acting and resource depletion [156,160,161]. Thirdly, the study also expands the social exchange theory in conjunction with the COR and JDR theories. It indicates that high LMX, characterized by strong interaction, mutual trust, respect, understanding, and communication between healthcare professionals and managers, can positively affect the influence of surface acting on stress and emotional exhaustion.

The study provides valuable insights and practical implications for organizations where health sector employees frequently engage in surface acting. It is found that high job demands can lead to negative workplace experiences such as stress and burnout when healthcare professionals engage in surface acting. This issue is crucial for a sustainable management approach and should receive attention from healthcare professionals and institutions from a public health perspective. In fact, healthcare professionals often overlook the adverse effects of emotional labor, and from a management and organizational standpoint, it is frequently deemed a job requirement and viewed as a performance criterion that must be endured [94]. However, this perspective is entirely wrong and triggers many negative situations for the individual and the organization. The main difference in this perspective can be better explained by how the individuals activate their internal mechanisms regarding the desired result or are expected to achieve. Stress differs from other experiences, such as anxiety, which shares similar symptoms with stress resulting from understandings such as employees’ performance expectations in the work environment; external factors usually trigger it, whereas anxiety is characterized by persistent, excessive worries even without a clear cause. Stress often arises from specific, identifiable events or situations such as work deadlines, conflicts, or ongoing challenges like chronic illness or discrimination [162]. Stress threatens the physical and psychological health of the individual by causing excessive cortisol production [163,164]. Some authors highlight the positive aspects of stress in the work environment and note that “eustress” can have a positive effect only with effective stress and job demands management. Otherwise, it can cause significant health and well-being problems [165]. Viewing high job demands (surface acting) as a success criterion in the workplace and considering it as an element that increases the performance of employees is a misunderstanding of elevated arousal. Elevated arousal can occasionally boost performance and concentration, known as the “Yerkes–Dodson Law”. However, a study that empirically examined the effect of the Yerkes–Dodson Law (YDL) on employees found that, contrary to expectations, it lacks empirical support. It suggests that the belief that increasing stress in the workplace will boost performance is a widely accepted misconception. The study also emphasizes that increasing or manipulating employee stress levels to enhance performance will negatively affect individuals and the organization [166]. Surpassing the optimal stress level can have detrimental effects, impair performance, and result in adverse consequences. Prolonged high levels of autonomic arousal can negatively impact health. In a study conducted on 1807 employees, work-related high arousal positive affect (HAPA) has a U-shaped association with basal cardiovascular activity. High-level arousal negatively affects individuals’ health [167]. Continuous exposure to high-stress hormones, particularly cortisol, may lead to conditions such as high blood pressure, heart diseases, and mental and physical health issues like depression, pain, burnout, and anxiety [168,169,170]. This can negatively impact the employee, the health organization, and the overall health system. The literature emphasizes that increasing employees’ awareness and understanding of events can prevent or mitigate these adverse outcomes [171]. Therefore, by improving the perception and perspective of healthcare professionals regarding the situation in which they have to display emotions that they do not feel for a long time during the surface-acting process, a depletion of their resources and experiencing emotional and cognitive dissonance can be prevented. First, this issue can be started by developing the personal capacities of healthcare professionals in healthcare organizations. Employee mindfulness and workplace buoyancy can reduce the stress and burnout that increase due to the negative situation employees experience within the framework of surface acting. A study in the literature has revealed that workplace buoyancy increases employees’ resources and ability to cope with difficulties, significantly reducing stress and emotional exhaustion in environments requiring high job demands [88]. Again, it is shared in the literature that surface acting experiences of individuals with high mindfulness ability decrease [39,172]. Employees’ resources can be improved by applying Mindfulness and Workplace Buoyancy-Based Stress Reduction programs to healthcare professionals, both during the start-up process and at certain periods, under the coordination of the human resources department. The effect of surface acting can be reduced through effective stress management by enabling the employee to reconsider their perspective on events and applying current proven methods such as yoga or stress management tactics within the scope of mindfulness and workplace buoyancy training. Secondly, resources within the organization can provide essential resources for healthcare professionals to balance job demands and resources. Among these resources within the organization, especially the perceived leadership approach and the supportive atmosphere created by the support of colleagues, can help reduce the pressure caused by surface acting on employees [173,174,175,176]. Implementing orientation training on a larger scale and over a more extended period, especially when healthcare professionals are new, will be an essential start for a supportive organizational climate. In this process, it would be appropriate to increase the knowledge and experience of healthcare professionals about how things work in different processes in the hospital environment to participate in meetings and demonstrate the accepted rules of behavior in the field. Again, to create a supportive organizational climate, the working environment’s physical facilities and personal development opportunities can be improved to facilitate healthcare professionals’ psychological and cognitive needs. In this way, the bonds that healthcare professionals establish with each other, and their organizations improve, and the emotional burden on them can be reduced somewhat thanks to the supportive organizational climate. Finally, the effect of surface acting on stress and burnout can be reduced through the relationship between manager healthcare professionals and team member healthcare professionals based on social exchange. At this point, health organizations should emphasize the perception of the LMX experience and make organizational improvements for a high LMX experience. First of all, regarding high LMX experience, it would be appropriate to organize practical training in the organization to adopt an open communication policy and disseminate it in practice. In addition, by determining the leadership perception experienced, leadership-development training programs can be organized for leaders to exhibit more positive leadership styles. In the literature, studies conducted specifically for healthcare professionals show that servant leadership contributes significantly to experiencing high LMX [177,178]. At this point, it may be essential to conduct the recruitment processes by considering “altruism, service orientation, ethics and integrity” behaviors [178]. In addition, in the previously emphasized leader-development training programs, applied training can develop the leadership abilities of healthcare managers who care about career development and can take action by accurately identifying their work-related needs [179]. In this way, within the framework of strong interpersonal relationships and social resources needed by healthcare professionals in daily processes, the effect of surface acting on stress and emotional exhaustion can be mitigated, and a healthy and robust organizational climate and management practice can be established.

## 8. Limitations and Future Directions

Although this study has some limitations, these limitations offer essential research areas for future studies. First of all, the cross-sectional design of the study limits causal interpretations. At this point, longitudinal and experimental studies examining the current model will increase the causality ability. Secondly, although the chief physicians of the hospitals operating in Ankara were interviewed, only two hospitals were permitted to conduct the study, and the data were obtained from these two hospitals using the convenience sampling method. The sample size of the study was relatively small. Future research might evaluate if the results of this study can be applied to different organizations and cultural settings by utilizing more extensive samples to extend our understanding of the effect of surface acting on stress and emotional exhaustion. Furthermore, the study mainly scrutinized the role of social exchange through the lens of Leader-Member Exchange (LMX). Future investigations could broaden this scope by considering other environmental factors such as the authenticity climate [39] and individual factors like emotion regulation self-efficacy [180], emotional intelligence [181], mindfulness, and workplace buoyancy [39,88]. These could potentially serve as buffering effects in the surface acting, stress, emotional exhaustion, and harmful interaction mechanisms.

Despite these limitations, the study findings underscore the significance of surface acting in healthcare organizations. The study provides insights into why this emotional labor strategy may lead healthcare professionals toward stress and emotional exhaustion. It also proposes solutions to counteract this negative mechanism that, in the long term, could undermine organizational effectiveness and employee health. Strong Leader-Member Exchanges (LMX) could be a potential mitigation strategy.

## Figures and Tables

**Table 1:** Demographic characteristics of the participants.

Variables		Frequency	%
Gender	Female	209	59.7
	Male	141	40.3
Marital Status	Married	225	64.3
	Single	125	35.7
Age	25 years and under	55	15.7
	26–35	122	34.9
	36–45	115	32.9
	45 years and above	58	16.6
Education	High school	55	15.7
	Associate Degree	84	24.0
	Bachelor Degree	157	44.9
	Post-Graduate	54	15.4
Profession	Physician	50	14.3
	Nurse or Midwife	118	37.7
	Technician/Technician	36	10.3
	Secretary	49	14.0
	Other	97	27.7

**Table 2:** Reliability and convergent validity analysis.

Variables	Factor Loadings	Cronbach Alpha	CR	AVE
Surface Acting	0.511–0.817	0.843	0.869	0.531
Job Stress	0.532–0.888	0.881	0.903	0.579
Emotional Exhaustion	0.522–0.862	0.806	0.814	0.548
Leader–Member Exchange	0.519–0.884	0.931	0.946	0.597

**Table 3:** Discriminant validity (Fornell-Larcker).

	1	2	3	4	5	6	Mean	sd.
1. Gender	1						-	-
2. Marital Status	0.102	1					-	-
3.Surface Acting	0.044	0.051	(0.729)				3.17	0.577
4. Job Stress	0.019 **	0.079 **	0.497 **	(0.761)			2.42	1.025
5. Emotional Exhaustion	−0.059	0.029	0.433 **	0.581 **	(0.740)		2.39	0.981
6. Leader–Member Exchange	0.175	0.098 **	−0.279 **	−0.357 **	−0.221 **	(0.773)	3.41	0.421

Note: Values in parentheses are Fornell–Larcker values. A statistically significant relationship was found between demographic variables, gender, and job stress (r = 0.019 **, *p* > 0.05). However, no relationship was detected with other variables. There was a positive correlation between marital status and job stress (r = 0.079 **, *p* > 0.05), as well as leader–member exchange (r = 0.098 **, *p* > 0.05). A statistically significant positive relationship was found between one of the research variables, surface acting, and both job stress (r = 0.497 **, *p* > 0.05) and emotional exhaustion (r = 0.433 **, *p* > 0.05). However, a statistically negative relationship was detected between leader–member exchange (r = −0.279 **, *p* > 0.05). Consequently, as the participants’ levels of surface acting increase, their levels of job stress and emotional exhaustion also increase.

**Table 4:** The moderator role of leader–member exchange.

	β	s.e.	t	p	LLCI	ULCI
**Independent variable: Job Stress**						
Surface Acting (X)	0.425	0.071	1.589	0.000	0.138	0.437
Leader–Member Exchange (W)	−0.271	0.069	−3.581	0.035	−0.191	−0.078
Interactional Term (X.W)	0.087	0.011	3.079	0.000	0.269	0.688
**Independent variable: Emotional Exhaustion**						
Surface Acting (X)	0.296	0.071	1.181	0.006	0.217	0.244
Leader–Member Exchange (W)	−0.171	0.054	−3.248	0.014	−0.222	−0.157
Interactional Term (X.W)	0.105	0.078	2.921	0.004	0.189	0.412

## Data Availability

The data supporting this study’s findings are available from the corresponding author upon reasonable request.
